# Retraction Note: A brief comparative examination of tangent hyperbolic hybrid nanofluid through a extending surface: numerical Keller–Box scheme

**DOI:** 10.1038/s41598-023-33083-5

**Published:** 2023-04-13

**Authors:** Wasim Jamshed, M. Prakash, S. Suriya Uma Devi, Rabha W. Ibrahim, Faisal Shahzad, Kottakkaran Sooppy Nisar, Mohamed R. Eid, Abdel‑Haleem Abdel‑Aty, M. Motawi Khashan, I. S. Yahia

**Affiliations:** 1 Department of Mathematics, Capital University of Science and Technology (CUST), Islamabad, 44000 Pakistan; 2 Department of Mathematics, Dr. N.G.P. Institute of Technology, Coimbatore, 641048 India; 3 Department of Mathematics, KPR Institute of Engineering and Technology, Coimbatore, 641407 India; 4 IEEE: 94086547, 59200 Kuala Lumpur, Malaysia; 5 Department of Mathematics, College of Arts and Sciences, Prince Sattam Bin Abdulaziz University, Wadi Aldawaser, 11991 Saudi Arabia; 6 Department of Mathematics, Faculty of Science, New Valley University, Al‑Kharga, 72511 Al‑Wadi Al‑Gadid Egypt; 7 Department of Mathematics, Faculty of Science, Northern Border University, Arar, 1321 Saudi Arabia; 8 Department of Physics, College of Sciences, University of Bisha, P.O. Box 344, Bisha, 61922 Saudi Arabia; 9 Physics Department, Faculty of Science, Al-Azhar University, Assiut, 71524 Egypt; 10 Department of Basic Sciences, Common First Year, King Saud University, Riyadh, 11451 Saudi Arabia; 11 Laboratory of Nano‑Smart Materials for Science and Technology (LNSMST), Department of Physics, Faculty of Science, King Khalid University, P.O. Box 9004, Abha, Saudi Arabia; 12 Research Center for Advanced Materials Science (RCAMS), King Khalid University, P.O. Box 9004, Abha, 61413 Saudi Arabia; 13 Nanoscience Laboratory for Environmental and Biomedical Applications (NLEBA), Metallurgical Lab. 1., Semiconductor Lab., Department of Physics, Faculty of Education, Ain Shams University, Roxy, Cairo, 11757 Egypt

Retraction of: *Scientific Reports* 10.1038/s41598-021-03392-8, published online 15 December 2021

The Editors have retracted this Article.

After publication concerns were raised about the originality of the work presented. Investigation has shown significant overlap with other sources, notably^1–3^. The Editors therefore consider this article to be redundant.

Wasim Jamshed and Kottakkaran Sooppy Nisar disagree with the retraction. S. Suriya Uma Devi, Rabha W. Ibrahim, Faisal Shahzad, Mohamed R. Eid, Abdel-Haleem Abdel-Aty, M. Motawi Khashan, and I. S. Yahia did not reply to the correspondence about the retraction. The Editors were not able to obtain a current email address for M. Prakash.
